# Editorial: Proceedings of KININ2018CLE, Cleveland, Ohio, June 18-20, 2018: A Compendium of the Presentations

**DOI:** 10.3389/fmed.2019.00272

**Published:** 2019-11-22

**Authors:** Alvin H. Schmaier, Marvin T. Nieman, Keith R. McCrae, Sadashiva Karnik, Joao Bosco Pesquero

**Affiliations:** ^1^Department of Medicine and Division of Hematology and Oncology, University Hospitals Cleveland Medical Center and Case Western Reserve University, Cleveland, OH, United States; ^2^Department of Pharmacology, Case Western Reserve University, Cleveland, OH, United States; ^3^Department of Cardiovascular and Metabolic Sciences and Hematology and Medical Oncology, Cleveland Clinic, Cleveland, OH, United States; ^4^Department of Cardiovascular and Metabolic Sciences, Cleveland Clinic, Cleveland, OH, United States; ^5^Department of Biophysics, Universidade Federal de São Paulo, São Paulo, Brazil

**Keywords:** bradykinin, factor XII, kallikrein, renin, angiotensin

## Introduction

KININ2018CLE was an international kinin meeting held on the campus of Case Western Reserve University, Cleveland, OH, USA from June 17th to 20th, 2018. This 3-day conference covered the gamut of topics related to the contact activation system, the kallikrein-kinin system, bradykinin biology, and interactions with the renin angiotensin system. This ebook was graciously complied by Frontiers in Medicine (Hematology) and serves as a Proceedings of the meeting. The Proceedings consists of 14 articles submitted by meeting participants. These articles are classified into four categories: contact activation; hereditary angioedema, C1 inhibitor, and bradykinin receptors; kallikreins, kinins, angiotensin converting enzyme; and renin-angiotensin. The papers within each of these four categories will be introduced in this editorial.

## Contact Activation

Mailer et al. presented a paper on polyphosphates (polyP) as a target for interference with inflammation and thrombosis. In their article they give an overview of polyP function, focusing on intra- and extracellular roles of the polymer and discuss open questions that emerge from current knowledge on polyP regulation. Conway discusses his observations of polyP on the complement system. Although polyP promotes factor XII activation, that is regulated by C1 inhibitor. In the complement system polyP potentiates C1 inhibitor and heparin inhibition of C1 activation and contributes to blocking the C5 convertase.

## Hereditary Angioedema, C1 Inhibitor, and Bradykinin Receptor Biology

Sanrattana et al. presented a concise review of SERPIN biochemistry and how serpin modification is useful to design new therapeutic tools. Veronez et al. presented a paper examining genetic variability of genes related to bradykinin formation and use to explain phenotypic variability of patient with hereditary angioedema (HAE). She also submitted a novel case report on an association of HAE with acute pancreatitis. Silva et al., presented an important report that bradykinin released by the erythrocytic stages of plasmodium falciparum enhances adhesion of infected red blood cells to endothelium to increase permeability via activation of bradykinin receptors. Perhal et al. from the Quitterer laboratory presented the fascinating finding that deficiency of the bradykinin B2 receptor protects mice from atherosclerosis. Last, Wu et al. from the laboratory of the late Marco Cicardi presented a brief report on a novel device to examine endothelial barrier function as it relates to angioedema and related disorders.

## Kallikreins, Kinins, and Angiotensin Converting Enzyme

Alhenc-Gelas et al., the 2018 Kinin Medal recipient, and his collaborators contributed an important paper on the evolving concept that although bradykinin formation is dependent on local tissue kallikrein and angiotensin converting enzyme (ACE) activity, deficiencies of BK result in detrimental effects of tissue kallikrein and ACE. Barros et al. who is in the Bader laboratory published a related observation that chronic overexpression of bradykinin in the kidney causes polyuria and cardiac hypertrophy. Campbell discussed the mechanisms of neprilysin and its inhibition in the treatment of heart failure and hypertension.

## Renin Angiotensin

Jara et al. summarize her work on murine tonin overexpression and how by diminishing sympathetic autonomic modulation by altering angiotensin type 1 receptor responses. Wolf et al. submitted an intriguing paper in which it was observed that sensitization of the angiotensin II AT1 receptor contributes to raf kinase inhibitor protein (RKIP)-induced symptoms of heart failure. Last, Quitterer and AbdAlla presented a brief summary of their seminal work in which pathologic co-localization of G-protein coupled receptors in hetero- or homo-dimers can contribute to disease states like pre-eclampsia.

In conclusion, these 14 articles are just a small sampling of the rich science presented at KININ2018CLE. The success of the meeting was in large part from the support we had from the National Institutes of Health, Division of Heart, Lung, and Blood Institute (R13HL140902), industry (Shire, CSL Behring, Alnylam, Kalvista, Enzyme Research Laboratories, Affinity Biologicals, and Diapharma) and a generous patient donor.

## Author Contributions

AS wrote the first draft of the manuscript. All authors reviewed and edited the manuscript.

### Conflict of Interest

The authors declare that the research was conducted in the absence of any commercial or financial relationships that could be construed as a potential conflict of interest.

